# Oral Health-Related Quality of Life among a large national cohort of 87,134 Thai adults

**DOI:** 10.1186/1477-7525-9-42

**Published:** 2011-06-13

**Authors:** Vasoontara Yiengprugsawan, Tewarit Somkotra, Sam-ang Seubsman, Adrian C Sleigh, The Thai Cohort Study Team

**Affiliations:** 1 National Centre for Epidemiology and Population Health, The Australian National University, Canberra, Australia; 2 Department of Community Dentistry, Faculty of Dentistry, Chulalongkorn University, Bangkok, Thailand; 3 School of Human Ecology, Sukhothai Thammathirat Open University, Nonthaburi, Thailand

**Keywords:** Oral Health-Related Quality of Life, oral health, tooth loss, cohort study, Thailand

## Abstract

**Background:**

Oral health has been of interest in many low and middle income countries due to its impact on general health and quality of life. But there are very few population-based reports of adult Oral Health Related Quality of Life (OHRQoL) in developing countries. To address this knowledge gap for Thailand, we report oral health findings from a national cohort of 87,134 Thai adults aged between 15 and 87 years and residing all over the country.

**Methods:**

In 2005, a comprehensive health questionnaire was returned by distance learning cohort members recruited through Sukhothai Thammathirat Open University. OHRQoL dimensions included were discomfort speaking, swallowing, chewing, social interaction and pain. We calculated multivariate (adjusted) associations between OHRQoL outcomes, and sociodemographic, health behaviour and dental status.

**Results:**

Overall, discomfort chewing (15.8%), social interaction (12.5%), and pain (10.6%) were the most commonly reported problems. Females were worse off for chewing, social interaction and pain. Smokers had worse OHRQoL in all dimensions with Odds Ratios (OR) ranging from 1.32 to 1.51. Having less than 20 teeth was strongly associated with difficulty speaking (OR = 6.43), difficulty swallowing (OR = 6.27), and difficulty chewing (OR = 3.26).

**Conclusions:**

Self-reported adverse oral health correlates with individual function and quality of life. Outcomes are generally worse among females, the poor, smokers, drinkers and those who have less than 20 teeth. Further longitudinal study of the cohort analysed here will permit assessment of causal determinants of poor oral health and the efficacy of preventive programs in Thailand.

## Background

Oral health is an important component of both overall health and quality of life. Oral disease creates a major public health burden worldwide and receives inadequate attention in many low and middle income countries [1]. Recently, particular attention is given to increasing the global awareness of the significance and inequity of oral health and the importance of its social determinants [2]. Oral diseases including oral cancers, periodontal disease, dental caries, and tooth loss are linked to emerging chronic non-communicable diseases primarily because of common risk factors such as poor dietary habits, poor oral hygiene, and use of tobacco and alcohol [3]. The joint effects of poor oral health and chronic diseases are major impediments to overall population health and quality of life, especially among the socioeconomically disadvantaged.

Oral Health-Related Quality of Life (OHRQoL) is defined by individual assessment of several oral health dimensions including physical dental function, tooth pain, psychological discomfort, and social impacts--all of which affect overall well-being [4-6]. Self-reported subjective indicators of OHRQoL correlate well with objective clinical measures of oral health status [4-8]. So OHRQoL at the individual level points to the need for clinical treatment and at the population level can be used to evaluate oral health interventions.

In the past decade, there have been several Asian studies on OHRQoL focused on adult populations in Hong Kong, Sri Lanka and Vietnam [9-11]. In Thailand, most population-based OHRQoL studies have focused on children because poor oral health at the early life can lead to a high lifelong impact [12]. Among Thai children, poor socioeconomic status has a powerful adverse effect on OHRQoL [13,14]. There are very few studies on adult OHRQoL in Thailand especially at the population-based level [15,16].

To fill the knowledge gap regarding OHRQoL among young and middle aged adults in Thailand, we included a broad oral health-related quality of life assessment in a large national Thai cohort study that began in 2005. Here we report the baseline distribution of OHRQoL among the cohort of 87,134 Thai adults; we investigate relationships with sociodemographic characteristics, health behaviours, and tooth loss. By linking OHRQoL status to its key determinants among such a large group of Thai adults, we generate evidence that can provide information on oral health goals for Thai adults and may incorporate the OHRQoL into oral health policy of the country in the future.

## Methods

### Study population and data collection

Data were derived from a cohort of 87,134 distance learning students aged 15 to 87 years enrolled at Sukhothai Thammathirat Open University (STOU) who completed a baseline study in 2005. The baseline characteristics of cohort participants compared to the population of Thailand have been reported [17]. The cohort represented well the main regions of Thailand and the modest income profile with a mean below US$3000 per year. There were a slight excess of females, with the overall median age of 29 years. The baseline questionnaire covered a wide range of topics including demographic, socioeconomic and geographic characteristics, health status, health service use, risk behaviours including smoking and drinking, injuries, dietary intake, physical activities, and family background. A four-year follow-up was conducted in 2009 and the next one is scheduled for 2013.

In the analysis presented here, individual characteristics analysed for association with OHRQoL include sex, age (15-29 years, 30-49 years, and 50 and older); income per month (less than 3000 Baht, 3001-7000 Baht, 7001-1000 Baht, and more than 10000 Baht: 40 Baht ~ US$1 in 2005); education (high school, diploma, university), and household assets (later categorized by total replacement value in Thai Baht into three groups ('low' < 30,000 'middle' 30,001-60,000 and 'high' > 60,000). The household assets included general domestic items such as a microwave oven, electric fan, air conditioner, computer, radio, video/vcd recorder, washing machine, water heater, and telephone.

As well, we determined lifecourse urbanization based on geographic residence now (as an adult member of the cohort) and when aged 12 years--creating the following urbanization categories (rural-rural or 'lifetime rural residents'; 'rural-urban' or 'rural-urban migrants'; and urban-urban or 'lifetime urban residents'). Since the cohort members are aged from 15 to 87 years, their lifecourse opportunities since age 12 vary accordingly. The small number of cohort members (4%) who were categorized as urban-rural were excluded from the analysis reported here so we could concentrate on the main categories that characterized the Thai population today. Health risk behaviors included in analyses were smoking (never, ever or regular current) as well as alcohol drinking (whether or not regular).

### Oral Health-Related Quality of Life (OHRQoL)

Impairment, functional limitation, disability, and handicap are pivotal concepts for the development of indicators for Oral Health-Related Quality of Life (OHRQoL). Locker [18] suggested a coherent theoretical framework of consequences of oral impacts, based on an adaptation of the WHO model for the International Classification of Impairments, Disabilities and Handicaps (1980) [19]. That is oral diseases can lead to impairment resulting in functional limitation, disability, and handicap [20].

Measurement of OHRQoL is a multidimensional process that incorporates incompletely demarcated domains such as illness, impairment, social, psychological and physical function and disability, oral health perceptions, as well as interactions between these domains. The application of OHRQoL indicators for each specific purpose may vary considerably. There are other measurement systems such as the General Oral Health Assessment Index (GOHAI), first used to assess clinical oral status and perceived impacts among the elderly [21]. A more recent system measures Oral Health Impact Profiles (OHIP) in 7 domains (functional limitation, physical disability, physical pain, psychological disability, psychological discomfort, social disability, and handicap) [22]. A related measure is the Oral Impacts on Daily Performances (OIDP) recording physical, psychological, and social difficulties [23,24].

We have adapted these concepts and measured negative impacts caused by oral health status. Questions asked were: "Do your teeth or dentures currently cause you?" (Multiple answers are allowed) Response categories include: 'discomfort speaking', 'discomfort swallowing', 'discomfort chewing', 'discomfort with social interaction', and/or 'pain'.

We have taken into account strong evidence in the literature that tooth loss is associated with OHRQoL and affects the severity of the impairment and we noted that self-report of the number of remaining teeth has commonly been used in the literature [25]. Hence we also included a question on remaining teeth as follows: "Adults can have up to 32 natural teeth. How many of your own teeth do you have?" So we counted total number of teeth regardless of their functional status. The responses were dichotomized as having '< 20' or '≥20' remaining teeth.

### Data processing and statistical analysis

Data scanning and editing were done using Thai Scandevet, SQL and SPSS software. For analysis we used Stata version 9. Individuals with missing data for analyses presented here were excluded so totals vary a little according to the information available. Missing data usually involved 1% or less of observations, however, our results were stable given the large size of our dataset. Bivariate analyses were followed by backward stepwise multivariate logistic regression with p set at < 0.05.

### Ethical considerations

Ethics approval was obtained from Sukhothai Thammathirat Open University Research and Development Institute (protocol 0522/10) and the Australian National University Human Research Ethics Committee (protocol 2004344). Informed written consent was obtained from all participants.

## Results

### Characteristics of the cohort members

There were 87,134 cohort members analysed (Table 1), with 54.7% being female. Slightly over 50% of cohort members were aged between 15 and 29 years and 2.5% aged over 50 years. Incomes ranged from less than 3,000 Baht per month (10.8%) to more than 10,000 Baht per month (33.9%). Nearly 50% had high school education, the rest had post high school diplomas or university degrees. Almost 43.3% reported being lifetime rural residents and 31.5% had moved from rural to urban areas since the age of 12 years.

**Table 1:** Attributes of cohort members in 2005

Cohort attributes N = 87,134	Overall (%)	Age groups (%)		
		
		15-29 yrs	30-49 yrs	50 yrs+
	**100.0**	53.6	43.9	2.5
**Socio-demographic characteristics**				
Males	**45.3**	37.9	52.8	71.9
Females	**54.7**	62.1	47.2	28.1
Income (Baht/month)*				
< 3000	**10.8**	16.1	4.6	4.7
3001-7000	**30.1**	41.7	17.3	8.9
7001-10000	**22.7**	26.1	19.5	7.3
> 10000	**33.9**	13.3	56.7	76.1
*Education*				
Up to high school education	**48.7**	48.8	48.5	50.5
Post high school diploma/certificate	**26.9**	31.3	22.2	16.4
University degree	**24.1**	19.7	29.0	32.6
*Household assets (Baht)*				
0-30,000	**40.4**	50.0	30.0	20.7
30,001-60,000	**30.5**	29.4	32.1	27.5
> 60,000	**28.6**	20.2	37.6	50.8
*Lifecourse urbanization*				
Lifetime rural residents	**43.3**	47.3	39.3	28.2
Rural-urban residents	**31.5**	29.9	33.3	32.6
Lifetime urban residents	**20.0**	18.2	21.0	29.5
**Health behaviours**				
*Smoking*				
Not a regular smoker	**70.1**	77.5	62.5	45.7
Ever smoker	**15.4**	10.3	12.1	10.7
A regular smoker	**9.8**	7.9	20.3	37.2
*Alcohol drinking*				
Not a regular alcohol drinker	**93.9**	95.8	91.7	89.7
A regular alcohol drinker	**4.8**	2.9	6.9	7.9
**Number of teeth**				
Remaining teeth ≥ 20	**96.7**	97.6	96.4	82.8
Remaining teeth < 20	**3.3**	2.4	3.6	17.2

* In 2005, 40 Baht ~ 1$US

Health risk behaviours such as regular smoking were reported by 9.8% of cohort members and 4.8% reported drinking alcohol regularly. For remaining teeth, 3.3% among cohort members reported less than 20.

### Oral Health-Related Quality of Life among cohort members

Five dimensions of Oral Health-Related Quality of Life were assessed (Table 2). Overall, discomfort chewing (15.8%), discomfort with social interaction (12.5%), and pain (10.6%) were the most commonly reported. The oldest group had almost double the proportion reporting discomfort speaking, swallowing and chewing; while younger groups reported more discomfort with social interaction. As well, the lowest income group was more likely than the highest income group to report discomfort with social interaction (13.6% vs 11.1%) and pain (12.0% vs 9.0%). However, no other pattern relating OHRQoL dimensions to either income or household assets emerge. The university educated group reported the least problems with chewing, social interaction and pain. Regular smokers and alcohol drinkers experienced adverse OHRQoL on all dimensions. Cohort members reporting less than 20 remaining teeth had much worse OHRQoL (e.g., 12.6% vs 2.0% had difficulty speaking and 3.4% vs 0.5% had difficulty swallowing).

**Table 2:** Oral Health-Related Quality of Life (OHRQoL) by cohort attributes

Cohort attributes	Oral Health-Related Quality of Life outcomes (%)*				
	
	Speaking	Swallowing	Chewing	Social	Pain
	**2.3**	**0.6**	**15.8**	**12.5**	**10.6**
**Socio-demographic characteristics**					
Males	2.5	0.8	16.3	12.1	10.1
Females	2.1	0.5	15.3	12.8	11.0
*Age groups (years)*					
15-29	1.8	0.5	12.2	12.6	11.4
30-49	2.7	0.7	19.3	12.4	9.6
50+	5.9	1.6	30.7	10.1	10.3
*Income (Baht/month)*					
< 3000	2.4	0.6	13.4	13.6	12.0
3001-7000	2.1	0.7	15.1	13.5	11.8
7001-10000	2.1	0.6	15.2	12.7	10.8
> 10000	2.7	0.6	17.7	11.1	9.0
*Education*					
Up to high school education	2.2	0.6	16.1	12.7	10.7
Diploma/certificate	2.3	0.7	16.2	12.9	11.2
University degree	2.5	0.5	14.8	11.5	9.5
*Household assets (Baht)*					
0-30,000	2.1	0.6	15.6	13.5	11.8
30,001-60,000	2.4	0.6	16.2	12.5	10.6
> 60,000	2.4	0.6	15.6	11.0	8.8
*Lifecourse urbanization*					
Lifetime rural residents	2.1	0.6	15.2	12.3	10.9
Rural-urban residents	2.3	0.6	16.2	12.3	10.8
Lifetime urban residents	3.2	0.7	18.3	15.5	11.0
**Health behaviours**					
*Smoking*					
Not a regular smoker	2.1	0.5	14.3	12.0	10.3
Ever smoker	3.0	0.9	19.2	13.2	10.9
A regular smoker	3.0	0.9	20.3	15.0	12.4
*Drinking*					
Not a regular alcohol drinker	2.3	0.6	15.4	12.4	10.5
A regular alcohol drinker	3.4	0.8	22.2	15.1	12.7
**Number of teeth**					
Remaining teeth ≥ 20	2.0	0.5	15.0	12.1	10.5
Remaining teeth < 20	12.6	3.4	40.2	25.8	15.5

*All figures displayed are proportions representing the percent of a given group with the OHRQoL outcomes.

### Bivariate and Multivariate analysis of Oral Health-Related Quality of Life

Bivariate analyses between OHRQoL and cohort characteristics (Table 3) reported odds ratios and p values. Notably, those aged 50+ had most difficulty speaking, swallowing, and chewing but were less likely to report difficulty with social interaction. Being a regular smoker, regular alcohol drinker, and having less than 20 teeth were all statistically associated with poor OHRQoL. The largest oral health effects (ORs exceeding 6.0) associated less than 20 remaining teeth with difficulties speaking and swallowing.

**Table 3:** Bivariate association of Oral Health-Related Quality of Life and cohort attributes

Cohort attributes	Oral Health-Related Quality of Life outcome (Odds Ratios)				
	
	Speaking	Swallowing	Chewing	Social	Pain
**Characteristics**					
Males	1	1	1	1	1
Females	**0.84**[p = 0.00]	**0.58**[p = 0.00]	**0.92**[p = 0.10]	**1.06**[p = 0.01]	**1.10**[p = 0.00]
*Age (years)*					
15-29	1	1	1	1	1
30-49	**1.56**[p = 0.00]	**1.40**[p = 0.00]	**1.74**[p = 0.00]	0.98[p = 0.47]	**0.82**[p = 0.00]
50+	**3.44**[p = 0.00]	**3.25**[p = 0.00]	**3.25**[p = 0.00]	**0.78**[p = 0.00]	0.89[p = 0.12]
*Income (Baht per month)*					
< 3000	0.93[p = 0.31]	1.06[p = 0.62]	**0.73**[p = 0.00]	**1.28**[p = 0.00]	**1.39**[p = 0.00]
3001-7000	**0.78**[p = 0.00]	1.16[p = 0.17]	**0.83**[p = 0.00]	**1.26**[p = 0.00]	**1.35**[p = 0.00]
7001-10000	**0.79**[p = 0.00]	1.06[p = 0.70]	**0.83**[p = 0.00]	**1.17**[p = 0.00]	**1.22**[p = 0.00]
> 10000	1	1	1	1	1
*Education*					
Up to high school	**0.89**[p = 0.04]	**1.34**[p = 0.01]	**1.12**[p = 0.00]	**1.14**[p = 0.00]	**1.17**[p = 0.00]
Diploma/certificate	0.93[p = 0.27]	**1.38**[p = 0.00]	**1.13**[p = 0.00]	**1.14**[p = 0.00]	**1.22**[p = 0.00]
University degree	1	1	1	1	1
*Household assets (Baht)*					
0-30,000	**0.87**[p = 0.01]	1.14[p = 0.22]	1.01[p = 0.60]	**1.28**[p = 0.00]	**1.41**[p = 0.00]
30,001-60,000	0.99[p = 0.93]	1.15[p = 0.22]	1.05 [p = 0.06]	**1.17**[p = 0.00]	**1.23**[p = 0.00]
> 60,000	1	1	1	1	1
*Lifecourse urbanization*					
Lifetime rural residents	1	1	1	1	1
Rural-urban residents	1.07[p = 0.21]	1.01[p = 0.91]	**1.08**[p = 0.00]	1.00[p = 0.97]	0.99[p = 0.57]
Lifetime urban residents	**1.23**[p = 0.01]	0.97[p = 0.82]	1.04[p = 0.16]	0.99[p = 0.74]	**0.84**[p = 0.00]
**Health behaviours**					
*Smoking*					
Not a regular smoker	1	1	1	1	1
Ever smoker	**1.48**[p = 0.00]	**1.72**[p = 0.00]	**1.45**[p = 0.00]	**1.21**[p = 0.00]	**1.07**[p = 0.03]
A regular smoker	**1.49**[p = 0.00]	**1.89**[p = 0.00]	**1.56**[p = 0.00]	**1.30**[p = 0.00]	**1.25**[p = 0.00]
*Alcohol drinking*					
Not a regular drinker	1	1	1	1	1
A regular drinker	**1.53**[p = 0.00]	**1.38**[p = 0.00]	**1.57**[p = 0.00]	**1.26**[p = 0.00]	**1.24**[p = 0.00]
**Number of teeth**					
Remaining teeth ≥ 20	1	1	1	1	1
Remaining teeth < 20	**7.14**[p = 0.00]	**6.90**[p = 0.00]	**3.78**[p = 0.00]	**2.51**[p = 0.00]	**1.55**[p = 0.00]

Multivariate logistic regression models for each of the five OHRQoL dimensions are shown in Table 3. Each model initially incorporated all variables shown with bivariate analyses (sociodemographics, behaviours, and number of teeth) and only those variables significant at 0.05 level were retained in the model. Females had more problems with chewing (OR = 1.26), social interaction (OR = 1.21) and pain (OR = 1.25). Those aged 50 years and older reported twice as much difficulty speaking, swallowing, and chewing. Having income less than 3,000 Baht per month associated with poor OHRQoL on all five dimensions. Ever or regular smoking and regular alcohol drinking were all associated with poor OHRQoL on most dimensions. Having less than 20 teeth had the strongest adverse association with OHRQoL especially for difficulty speaking (OR = 6.43) and difficulty swallowing (OR = 6.27).

## Discussion

Our analysis of Oral Health-Related Quality of Life (OHRQoL) among a large cohort of Thai adults contributes to the growing population-based literature. We found that discomfort chewing, discomfort with social interaction, and pain were the most common oral impacts, affecting 10-16% of the respondents. Being of old age, having a very low income, (ever or current) smoking, and regular alcohol drinking were all associated with adverse oral impacts. Compared to the adverse effects on speaking and swallowing attributable to certain sociodemographic or behavioral characteristics, having < 20 remaining teeth was even more detrimental.

We used standard methods to de-confound our effect measures and produced adjusted odds ratios; however, some effects were quite small (e.g., less than 1.10) but still statistically significant due to the large sample size. Such small effects are reported but are not considered to be important from a public health point of view. Also, we noted that small bivariate effects with odds ratio close to 1 were sometimes reversed to the other side of 1 when adjusted in the multivariate analysis. Furthermore, certain explanatory variables were dropped by the stepwise process of multivariate logistic regression as a consequence of collinearity with another explanatory variable already in the model. For example, sex and household assets are dropped from the final multivariate model for speaking (Table 4). We also noted that there is probably some interaction occurring among various explanatory variables such as smoking, drinking and age but we did not attempt to model these effects separately.

**Table 4:** Multivariate association between Oral Health-Related Quality of Life* and cohort attributes

Attributes	Oral Health-Related Quality of Life (Adjusted Odds Ratios)				
	
	Speaking	Swallowing	Chewing	Social	Pain
**Characteristics**					
Males		1	1	1	1
Females		**0.69**[p = 0.00]	**1.26**[p = 0.00]	**1.20**[p = 0.00]	**1.25**[p = 0.00]
*Age (years)*					
15-29	1	1	1	1	1
30-49	**1.52 **[p = 0.00]	**1.53**[p = 0.00]	**1.77**[p = 0.00]	**1.09**[p = 0.00]	**0.92**[p = 0.00]
50+	**2.02 **[p = 0.00]	**2.22**[p = 0.00]	**2.85**[p = 0.00]	**0.77**[p = 0.00]	0.99[p = 0.90]
*Income (Baht per month)*					
< 3000	**1.40 **[p = 0.00]	**1.56**[p = 0.01]	1.02 [p = 0.57]	**1.30**[p = 0.00]	**1.31**[p = 0.00]
3001-7000	**1.16 **[p = 0.04]	**1.70**[p = 0.00]	**1.09 **[p = 0.00]	**1.26**[p = 0.00]	**1.20**[p = 0.00]
7001-10000	1.07 [p = 0.45]	**1.39**[p = 0.01]	1.03 [p = 0.38]	**1.17**[p = 0.00]	**1.13**[p = 0.00]
> 10000	1	1	1	1	1
*Education*					
Up to high school	**0.82**[p = 0.00]	1.04[p = 0.72]	**1.09**[p = 0.00]		0.99[p = 0.79]
Diploma/certificate	0.98[p = 0.80]	**1.33**[p = 0.03]	**1.18 **[p = 0.00]		1.07[p = 0.08]
University degree	1	1	1		1
*Household assets (Baht)*					
0-30,000			**1.20**[p = 0.00]	**1.24**[p = 0.00]	**1.27**[p = 0.00]
30,001-60,000			**1.14**[p = 0.00]	**1.16**[p = 0.00]	**1.17**[p = 0.00]
> 60,000			1	1	1
*Lifecourse urbanization*					
Lifetime rural residents	1		1	1	1
Rural-urban residents	1.10[p = 0.09]		**1.07**[p = 0.00]	**1.06**[p = 0.00]	1.05[p = 0.07]
Lifetime urban residents	**1.40**[p = 0.01]		1.01[p = 0.64]	**1.07**[p = 0.00]	**0.93**[p = 0.03]
**Health behaviours**					
*Smoking*					
Not a regular smoker	1	1	1	1	1
Ever smoker	**1.30**[p = 0.00]	**1.31**[p = 0.04]	**1.36**[p = 0.00]	**1.25**[p = 0.00]	**1.25**[p = 0.00]
A regular smoker	**1.32**[p = 0.00]	**1.44**[p = 0.01]	**1.51**[p = 0.00]	**1.39**[p = 0.00]	**1.40**[p = 0.00]
*Alcohol drinking*					
Not a regular drinker	1		1	1	1
A regular drinker	**1.23 **[p = 0.04]		**1.26**[p = 0.00]	**1.24**[p = 0.00]	**1.27**[p = 0.00]
**Number of teeth**					
Remaining teeth ≥ 20	1	1	1	1	1
Remaining teeth < 20	**6.43**[p = 0.00]	**6.27**[p = 0.00]	**3.26**[p = 0.00]	**2.61**[p = 0.00]	**1.54**[p = 0.00]

Table displays results of one model for each of the five OHRQoL dimensions. Each model is based on backward stepwise multivariate logistic regression predicting the yes-no outcome for each of the five dimensions (Stata 10). Variables shown in each model were those retained as statistically significant at p < 0.05.

Our findings were generally consistent with a previous study in Thailand [16]. Of particular note is the adverse consequences of oral diseases on daily life including in the psychosocial dimension: feeling embarrassed in social settings, especially for females. Another report for Thai adults revealed that those of low socioeconomic status, with poor oral health-related behaviors such as smoking, and older age were more likely to self-report worse oral health and associated poor quality of life [15]. Another Asian report is notably consistent with the data from Thailand; Indian adults responding to a general health questionnaire reported that poor oral health had an important adverse effect on psychological distress [26].

Individual perceptions of oral health vary substantially at different points in the life cycle and cumulative risks could subsequently impact on later years [27,28]. We found an increasing gradient between age and difficulty speaking, swallowing and chewing with progressive deterioration for middle- and older-aged groups. One of the main domains of OHQRL noted in literature was the difficulty chewing, especially among the elderly [29,30] which could result in limited choices of food, poor nutrition, and subsequently underweight [31]. In addition, poor oral health and under nutrition in older people could increase the incidence of life-threatening conditions, such as atherosclerosis and cancer [32]. In Thailand, a community dental study of Thai elderly has shown a strong association between number of natural teeth and being underweight after controlling for sex, socioeconomic status and current smoking (OR = 2.27, 95% CI 1.25-4.13) [33]. Using the Oral Impact on Daily Performance indicators, another Thai study among elderly showed significant associations between oral impacts and a variety of clinical measures [34]. More specifically, edentulous people experience higher levels of impacts in comparison to the dentate, especially notable for pain, chewing, and nutrition.

The strength of our study is its large national scale with nearly 90,000 adults representing the Thai population well in terms of socioeconomic and geographic background in Thailand. Our study is based on educated Thais and the true magnitude of the poor oral health will be even greater in the general population as reported in other studies that show an education gradient in OHRQoL, with worse outcome at lower levels of education [35]. Outcomes and findings derived from self-reported research may vary in cross-cultural context which could impact on the measured health-related quality of life [36,37].

It should also be noted that our analyses depended on cross-sectional data so it was difficult to definitively establish causal associations. Furthermore, we could not link reported outcomes to dental care and prevention, nor could we assess the utility of early diagnosis and completion of treatment. Our Thai cohort could potentially shed light on the causal pathway between OHRQoL and other health outcomes when it becomes possible to make longitudinal analyses across adequate time spans. In addition, OHRQoL deserves particular attention in national health surveys.

Oral health quality deserves to be promoted in the national oral health plan to meet the needs of the population and achieve the optimal benefits from available resources. OHRQoL can measure the effectiveness of dental public health programmes, assessing the oral health needs of populations. At the population level, tailored strategies for different groups such as the elderly, low income persons, smokers, and people with less than 20 teeth will yield maximum benefit within limited resources. As revealed in our study, OHRQoL is a significant component of overall health among the whole population. The goal of oral health goes beyond the prevention of oral diseases. Oral health-related quality of life converges well with the holistic 1948 World Health Organization definition of health as 'complete physical, mental and social well-being and not merely the absence of disease'.

## Competing interests

The authors declare that they have no competing interests.

## Authors' contributions

VY and TS conceptualised, analysed and drafted the manuscript. SS and AS designed the project and helped to finalise the manuscript. The Thai Cohort Study team has contributed to all stages of the project. All authors read and approved the final manuscript.
